# Hierarchically Porous Carbon Derived from Biomass Reed Flowers as Highly Stable Li-Ion Battery Anode

**DOI:** 10.3390/nano10020346

**Published:** 2020-02-18

**Authors:** Weimin Zhao, Jingjing Wen, Yanming Zhao, Zhifeng Wang, Yaru Shi, Yan Zhao

**Affiliations:** 1 School of Materials Science and Engineering, Hebei University of Technology, Tianjin 300401, ChinaJingjingVane@163.com (J.W.); zhaoyanming@hebut.edu.cn (Y.Z.); 2 Key Laboratory for New Type of Functional Materials in Hebei Province, Hebei University of Technology, Tianjin 300130, China; 3 School of Mechanical Engineering, Hebei University of Technology, Tianjin 300401, China

**Keywords:** biomass, porous, carbon, Li-ion battery, anode

## Abstract

As lithium-ion battery (LIB) anode materials, porous carbons with high specific surface area are highly required because they can well accommodate huge volume expansion/contraction during cycling. In this work, hierarchically porous carbon (HPC) with high specific surface area (~1714.83 m^2^ g^−1^) is synthesized from biomass reed flowers. The material presents good cycling stability as an LIB anode, delivering an excellent reversible capacity of 581.2 mAh g^−1^ after cycling for 100 cycles at a current density of 100 mA g^−1^, and still remains a reversible capacity of 298.5 mAh g^−1^ after cycling for 1000 cycles even at 1000 mA g^−1^. The good electrochemical performance can be ascribed to the high specific surface area of the HPC network, which provides rich and fast paths for electron and ion transfer and provides large contact area and mutual interactions between the electrolyte and active materials. The work proposes a new route for the preparation of low cost carbon-based anodes and may promote the development of other porous carbon materials derived from various biomass carbon sources.

## 1. Introduction

With the excessive consumption of nonrenewable resources, the energy crisis spreads around the world. Therefore, it is urgent to explore new ways for storing energy [1,2]. As one of effective energy-storage devices, lithium ion batteries (LIBs) have attracted broad attention due to its high energy density, long cycling life, and light weight [3,4,5]. The property of an anode material has a significant effect on the final performances of a total LIB. At present, anode materials mainly include conversion reaction type, alloy-type, and carbonaceous materials [6,7,8,9]. However, the applications of both conversion-reaction-type and alloy-type anodes are restricted by their low conductivity and serious volume expansion during cycling [10,11,12,13]. Compared with these kinds of anodes, carbon-related materials (such as graphite, graphene, activated carbon, carbon nanotubes, carbon fibers, etc.) present good characteristics including high safety, high conductivity, low cost, excellent electrochemical stability, and high mechanical firmness [14,15,16,17,18,19]. Therefore, carbon materials have become mainstream materials for commercial LIB anodes for many years. In particular, porous carbon materials with plenty of network structure can provide short diffusion pathways for ion and electron transport and large active regions for electrochemical reactions [20,21,22,23]. In this situation, the preparation methods of porous carbon materials were studied by scientists from all over the world with great enthusiasm. Among various ways, it is a green and environmentally friendly route to fabricate porous carbon from biomass wastes [24,25]. In recent years, the synthesis methods towards porous carbon derived from different biomass materials have been developed successfully. Many biomasses such as walnut shell, duckweed, coir pith, and bean-dreg [26,27,28] are developed into porous carbon materials. These materials show good electrochemical performance as anodes for LIBs. However, it is difficult to collect some biomass materials in a relatively concentrated region, which limits the mass production of biomass carbon materials.

As we know, reed widely grew near lakes and rivers, so that reed flowers can be collected easily, which provides abundant feedstock to prepare biomass carbons with unique morphologies for large-scale production. As the starting materials, reed flower possesses the following advantages compared with other biomass materials. Firstly, compared with other biomasses such as dandelion, loofah, and jute [29,30], reed flowers can be collected in a larger area. In special, higher harvesting quality can be obtained per unit time. In addition, compared with biomasses [31,32] such as banana peel, prunus persica, and coconut shell, the natural fluffy structure of reed flower is beneficial to the synthesis of porous carbon materials with high specific surface area. Moreover, compared with a large number of edible biomass carbon sources [33,34], reed flowers can be utilized directly and do not need to wait after they finish the edible value. In this study, we fabricate hierarchically porous carbon (HPC) with high specific surface area from reed flowers by a multistep calcination method. When the HPC is applied to anode materials for LIBs, it exhibits good cycling capability and rate performance. The paper provides us an idea for developing low-cost porous carbon materials derived from biomass and may promote the development of porous biomass carbon-based materials in energy-storage applications.

## 2. Materials and Methods

### 2.1. Material Preparation

**Figure 1:**
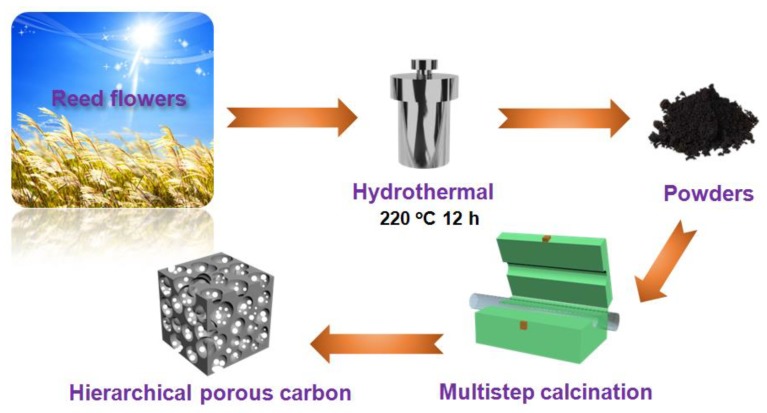
Schematic illustration of the synthesis procedure for the preparation of hierarchical porous carbon (HPC).

The HPC was obtained from biomass reed flower. The typical synthesis process is shown in Figure 1. Firstly, 40 mL deionized water (DI-water) was introduced into the 100 mL Teflon-lined autoclave, and then, 1 g reed flowers was dispersed in the deionized water. The mixture was heated to 220 °C for 12 h. When the Teflon-lined autoclave cooled to 25 °C, the hydrothermally synthesized product was taken out and centrifugally separated with DI-water. After the products were dried in a drying oven at 80 °C for 10 h, black powders were obtained. Then, 1 g powders and 1 g KOH were mixed evenly and added into 20-mL DI-water with magnetic stirring for 1 h. The mixture was dried at 80 °C for 10 h. In the next step, the compound was multistep calcinated at different temperatures (450 °C for 30 min, 650 °C for 30 min, and 800 °C for 1 h) under Ar protection with a heating rate of 5 °C min^−1^, so that the products were fully activated and carbonized, creating plenty of pores in different sizes. After the furnace cooled to 25 °C, the products were washed by 10% HCl for 10 min and then washed by DI-water for 5 times. At last, the products were dried at 80 °C for 12 h for further testing.

### 2.2. Characterization

The phase of as-prepared samples was analyzed by X-ray diffraction (XRD, Rigaku-TTRIII, Tokyo, Japan) using Cu Kα radiation. The Raman spectra was identified on a Lab RAM HR800 (Horiba, Kyoto, Japan) with a 632-nm laser. Scanning electron microscopy (SEM, Hitachi S-4800, Tokyo, Japan) and transmission electron microscopy (TEM, JEM 2100, Tokyo, Japan) were used to characterize the microstructure and morphology of the sample. Nitrogen adsorption-desorption isotherm was performed on V-Sorb 2800P (Jinaipu, Beijing, China). The specific surface was analyzed by the Brunauer–Emmett–Teller (BET) method, and the Barrett–Joyner–Halenda (BJH) method was performed to obtain pore size distributions.

### 2.3. Electrochemical Measurements

Seventy percent of the HPC materials, 20% of the Ketjen black, and 10% of the carboxymethyl cellulose (CMC) binder was mixed in DI-water to form slurry. Then, the anode was fabricated by painting the slurry on Cu foil and was dried at 60 °C for 10 h. The HPC anode, lithium cathode, celgard 2400 separator, and the electrolyte (1 M LiPF_6_ in a mixed solution of ethylene carbonate and diethyl carbonate, EC/DEC = 1:1 by volume) were encapsulated into 2025 coin cells in a glovebox under an Ar atmosphere (H_2_O < 0.01 ppm and O_2_ < 0.01 ppm). The electrochemical impedance spectroscopy (EIS) and cyclic voltammetry (CV) of the batteries were performed on an electrochemical workstation (Princeton, VersaSTAT 4, Oak Ridge, TN, USA) at 0.1 mV s^−1^ from 0.01 to 3 V. Galvanostatic charge-discharge were tested on a NEWARE battery tester (Shenzhen, China) in the voltage window of 0.01–3 V (vs. Li^+^/Li).

## 3. Results and Discussion

**Figure 2:**
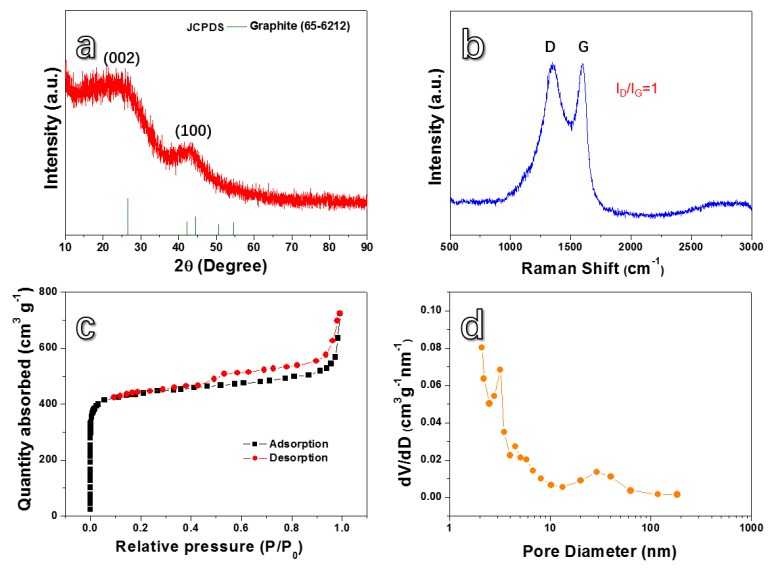
(**a**) X-ray diffraction (XRD) pattern, (**b**) Raman spectrum, (**c**) N_2_ adsorption-desorption isotherm characteristics, and (**d**) pore size distribution of the HPC.

Figure 2a shows the XRD patterns of the HPC. Two broad peaks at about 26° and 44° can be clearly found. The broad peak at 26° is ascribed to the (002) crystal plane of graphite (JCPDS No. 65-6212), while the peak around 44° relates to the (100) crystal plane of sp^2^-hybridized carbon [35,36,37]. These results demonstrate that the as-prepared HPC possess a graphitic structure [38,39,40]. Figure 2b presents Raman spectroscopy of the HPC material. Two peaks at ~1342 cm^−1^ and ~1599 cm^−1^ correspond to the D band and G band, respectively [30,31]. The D band represents defect and disorder [41,42], while the G band corresponds to the existence of SP^2^-hybridized carbon [43]. The intensity ratio of D to G is 1.0, which suggests that the HPC possesses a large number of defects and good electrical conductivity [44,45]. These characteristics are good for electron transfer and rate performance [46].

In order to evaluate the specific surface area and porosity of the HPC, nitrogen adsorption-desorption isotherms and the pore size distributions curves were measured and shown in Figure 2c,d, respectively. As shown in Figure 2c, the curve displays a typical type-IV adsorption-desorption isotherm with a type-H4 hysteresis loop [38], demonstrating the existence of both micropores and mesopores. A relatively high specific surface area of ~1714.83 m^2^ g^−1^ and a big pore volume (~1.13 cm^3^ g^−1^) are obtained based on the BET test. These data are much greater than those of other biomass carbon material [26,27,28,38]. It can be seen from Figure 2d that the pore sizes of the HPC mainly distribute between 2 and 5 nm, presenting obvious mesoporous characteristics. These mesopores, as second-class pores, together with the first-class pores (macropores, ~70 nm), form a hierarchically porous structure. The large specific surface area makes the electrolyte–electrode interface large enough to accumulate rich charges and ions [47,48]. Moreover, the hierarchically porous structure can provide a fast channel for the migration of Li ions and electrons and provides enough space for the volume expansion/contraction during cycling to obtain a stable Li storage [49].

**Figure 3:**
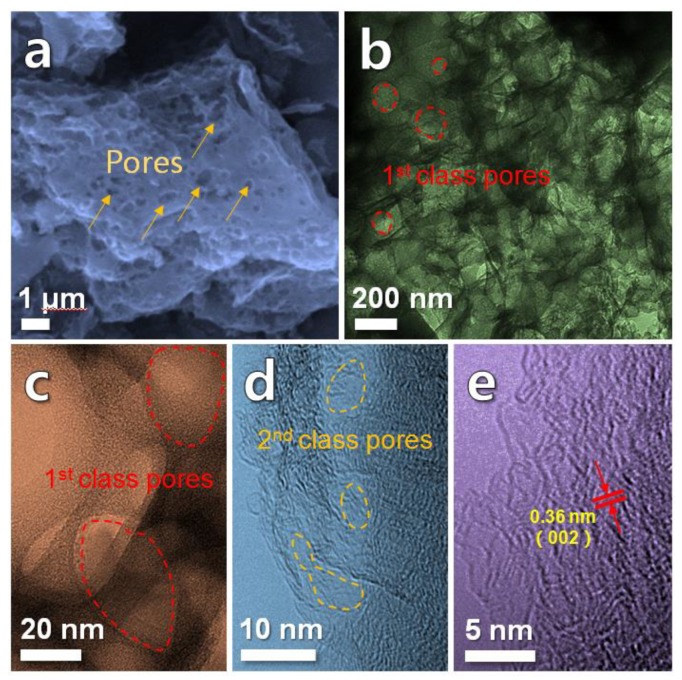
(**a**) Scanning electron microscopy (SEM) image of HPC and (**b**–**e**) transmission electron microscopy (TEM) images of HPC. Red circles marked in (**b**) and (**c**) show first class pores. Yellow circles marked in (**d**) show second class pores. Arrows in (**e**) mark the lattice fringe spacing.

Figure 3a shows a SEM image of the HPC. It is clearly shown that the HPC exhibits a typically porous structure with rich macropores (a few hundred nanometers in diameter). Figure 3b–e presents TEM images of the HPC. A large number of pores (tens to hundreds of nanometers in diameter) can be found in Figure 3b,c, forming the first class pores. Plenty of mesopores with a diameter of a few nanometers can be seen in Figure 3d, constituting the second-class pores. In this situation, a hierarchical porous structure containing the first-class pores (macropores) and the second-class pores (mesopores) is successfully obtained. This result is coincidence with the pore size distribution result (Figure 2d). Furthermore, the lattice fringe spacing of 0.36 nm marked in Figure 3e is consistent with the (002) planes of the graphite (JCPDS No. 65-6212), corresponding well with the XRD result.

**Figure 4:**
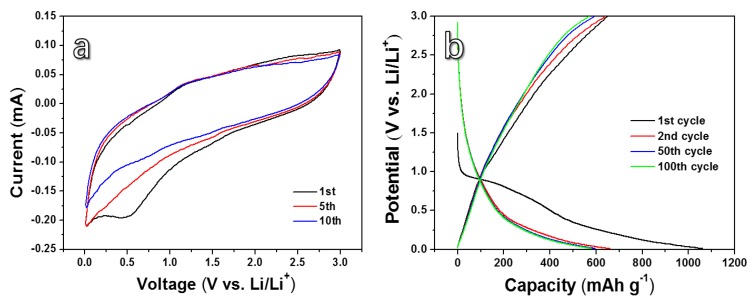
(**a**) Cyclic voltammetry (CV) curves measured at 0.1 mV s^−1^ between 0.01–3 V and (**b**) galvanostatic charge-discharge (GCD) curves measured under 100 mA g^−1^ for the lithium-ion battery (LIB) device with the anode material of HPC.

CV curves (Figure 4a) towards the HPC anode were measured at a scan rate of 0.1 mV s^−1^ in the voltage range of 0.01–3 V. A broad reduction peak is found in the first cycle, suggesting the formation of the solid electrolyte interface (SEI) film on the HPC electrode surface [50]. An obvious cathodic peak between 0.01 and 2.0 V can be found, corresponding to reversible insertion of lithium into the carbon layers and nanopores [51]. The intensity of the above cathodic peak in the first loop is much higher than that of the rest, which may be due to the formation of the SEI, the electrolyte decomposition, and the irreversible insertion of Li^+^ into special sites of carbon material [52]. Figure 4b shows the discharge and charge curves from the 1st to 100th cycle of the HPC anode under a current density of 100 mA g^−1^ in a voltage range of 0.01–3 V. The HPC anode presents the 1st discharge and charge capacities of 1062.5 and 649.2 mAh g^−1^, respectively. The primary coulombic efficiency of HPC anode is about 61.1%. The irreversible capacity loss could be attributed to the formation of SEI, the irreversible lithium-ion intercalation on the new-formed surfaces, and even disordered carbon binder [53,54,55,56]. In the first discharge curve, a plateau is found at about 0.5 V and then disappears in the subsequent discharge curves. This phenomenon is also in line with the CV result. The discharge and charge capacities of the 2nd cycle are 662.5 and 641.7 mAh g^−1^, respectively, and the coulombic efficiency increases to 97.7%. Compared with the 50th and 100th discharge and charge curves, they almost overlap with each other, showing an outstanding cycling stability. 

**Figure 5:**
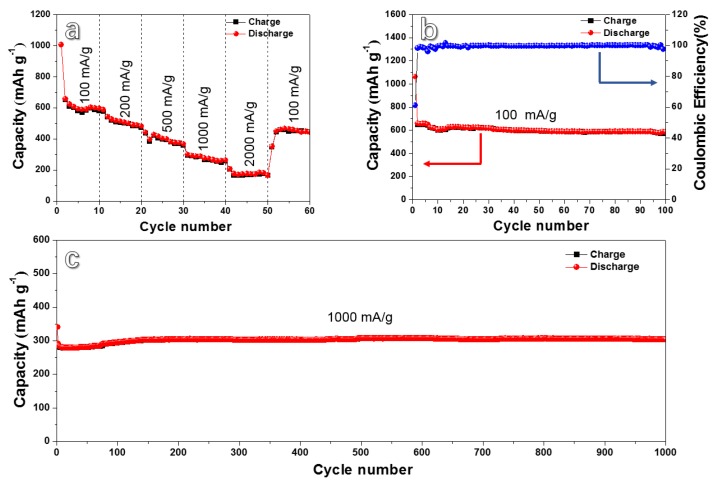
(**a**) Rate performance at various current densities and cyclic performance of HPC at current density of 100 mA g^−1^ (**b**) and 1000 mA g^−1^ (**c**).

The rate property of the HPC anode is shown in Figure 5a. Reversible capacities of 604, 512, 399, 279, and 175 mAh g^−1^ are achieved at different current densities from 100 to 2000 mA g^−1^. When the current density comes back to 100 mA g^−1^, the specific capacity returns to 460 mAh g^−1^, revealing a goodish rate performance. To further improve the rate performance, some previous reported methods, including nitrogen/phosphorus doping, conductive metal doping, and forming a three-dimensional conductive cross-linked carbon network, can be adopted [29,57,58,59]. The related work can be carried out in the future. The HPC electrode also exhibits good cycling performances (Figure 5b). When cycled for 50 cycles, the reversible capacity of the HPC anode reaches 593.4 mAh g^−1^. After cycling for 100 cycles, the reversible capacity of the HPC anode can be stabilized at 581.2 mAh g^−1^. Even cycling at a high current density of 1000 mA g^−1^, the HPC anode shows a discharge capacity around 300 mAh g^−1^ invariably, revealing an outstanding cycling stability. After 1000 cycles, a reversible capacity of 298.5 mAh g^−1^ still remains (Figure 5c). The good rate and cycling performance could be ascribed to the unique hierarchical porous structure of the biomass carbon material.

**Figure 6:**
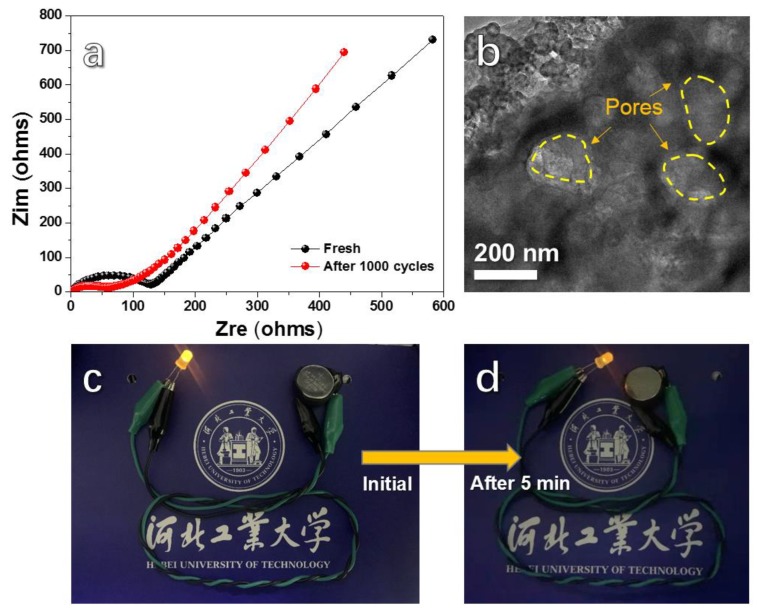
(**a**) Nyquist plots for HPC anode before and after 1000 cycles; (**b**) SEM image of HPC anode after cycling at 1000 mA g^−1^ for 1000 cycles; and digital photographs of a yellow LED bulb propelled by an HPC battery: (**c**) Initial and (**d**) after 5 min.

Figure 6a displays the EIS of the HPC anodes before and after cycling at 1000 mA g^−1^ for 1000 cycles. The semicircles in the high-frequency region are contact with the interface charge-transfer process, and the straight lines in the low-frequency region correspond to the Warburg diffusion inner the networks [48,49]. It can be found that the semicircle of the electrode becomes smaller and that the slope of the straight line becomes larger after 1000 cycles, which is due to the dissolution and redistribution of the active material. The SEM image of the HPC anode after 1000 discharge/charge cycles at 1000 mA g^−1^ is shown in Figure 6b. Many macropores maintain their initial shapes and sizes after cycling at such a high current density, which is favourable for obtaining good cycling stability. Figure 6c,d presents digital photographs of a yellow light emitting diode (LED) bulb propelled by an HPC half battery. The bulb darkens gradually. After 5 min, the brightness of the LED bulb decreases obviously compared with its initial state, while the bulb still can work.

Table 1 [27,28,38,44,45,46,53,60,61,62] compares the electrochemical performance of the current study with the previously reported works. It can be seen that the electrochemical performance of the HPC anode is superior to most of reported biomass-based carbon materials. The outstanding electrochemical property can be ascribed to the following aspects. Firstly, the HPC with high specific surface area can make the electrolyte–electrode interface large enough to accumulate rich charges and ions. In addition, it also can provide large contact area and mutual interactions between the electrolyte and active materials. Secondly, the first-class pores in the hierarchically porous structure can relieve the volume change during the cycling procedure while the second-class pores can provide rich and fast paths for the transfer of ions and electrons. Thirdly, the value of I_D_/I_G_ is 1.0, indicating that the materials contain both defects and good electrical conductivity. The defects are good for electron transfer, and the great electrical conductivity is beneficial to improve the rate property of the battery. Based on the above discussions, we can conclude that the HPC synthesized from biomass material in this study has great potential as anodes for LIBs. Moreover, this paper also provides us a new proposal for the fabrication of low-cost carbon-based anode materials, and the route is expected to be extended and applied to various biomasses in future.

## 4. Conclusions

Hierarchical porous carbon (HPC) was successfully synthesized by multistep calcination of biomass reed flowers in this work. The HPC anode exhibits a high specific surface of ~1714.83 m^2^ g^−1^ and a big pore volume (~1.13 cm^3^ g^−1^). Owing to the advantages of the hierarchically porous structure, the HPC electrode displays good electrochemical property with a discharge capacity of 581.2 mAh g^−1^ at 100 mA g^−1^ after 100 cycles, allowing for a promising prospect of further practical application in the battery industry as one of the effective and cheap alternatives of graphite. Moreover, the HPC anode achieves good rate performance. Even at a large current density of 1000 mA g^−1^, the specific capacity could also retain at 298.5 mAh g^−1^ after 1000 cycles. The above property of the as-fabricated HPC reveals its great potential as an LIB anode. This work not only visualizes the possible application of biomass carbon as an anode material for LIBs but also proposes a new route for the preparation of low-cost carbon-based anodes and may promote the development of other porous carbon materials derived from various biomass carbon sources.

## Figures and Tables

**Table 1:** Comparison of electrochemical performances for various biomass-derived carbons as LIB anodes.

Biomasses (Corresponding Carbon Materials)	Current Density (mA g^−1^)	Cycle Number	Reversible Capacity (mAh g^−1^)	Ref.
Coir pith waste (porous carbon)	100	50	837	[27]
Bean-dreg (graphitic sheet)	100	100	396	[28]
Waste green tea (graphitic carbon nanoflakes)	100	100	400	[38]
Rice husks (carbon-decorated silicon spheres)	100	100	429	[44]
Coffee waste (nonporous carbons)	100	100	285	[53]
Wheat flour (carbon particles)	1000	100	217	[45]
Orange peel (porous carbon)	1000	100	301	[46]
Loofah (three-dimensional porous carbon framework)	100	100	225	[60]
Waste green tea (nanoparticles)	100	100	498	[61]
walnut shell (porous carbon nanofiber)	100	200	280	[62]
Reed flowers (hierarchical porous carbon)	100 1000	100 1000	581.2 298.5	This work
